# Radiation of parotid or submandibular glands is effective for drooling in patients with parkinsonism; a randomised double-blind placebo-controlled trial

**DOI:** 10.1016/j.prdoa.2022.100138

**Published:** 2022-02-14

**Authors:** R.J.H.M. Steenbakkers, S.P. van Doornik, A. Vissink, W. Kerdijk, T. van Laar

**Affiliations:** a Department of Radiotherapy, University of Groningen, University Medical Center Groningen, the Netherlands; b Department of Dental Medicine, University of Groningen, Groningen, the Netherlands; c Department of Maxillofacial Surgery, University of Groningen, University Medical Center Groningen, the Netherlands; d Department of Neurology, University of Groningen, University Medical Center Groningen, Hanzeplein 1, 9700 RB Groningen, the Netherlands

## Abstract

**Background:**

Drooling is a common symptom in patients with parkinsonism, causing physical and emotional distress. It is unknown which major salivary glands are the best candidates for irradiation to reduce drooling with minimal adverse events. Therefore, this study assessed the efficacy and safety of submandibular and parotid salivary gland irradiation to reduce drooling.

**Methods:**

A prospective, randomised, double-blind, placebo-controlled trial was conducted at the University Medical Center Groningen, the Netherlands. After informed consent, 31 patients with parkinsonism and severe drooling according to the Unified Parkinson Disease Rating Scale (UPDRS) were included in this study. Exclusion criteria consisted of the use of anticholinergic drugs, the existence of salivary gland diseases, and/or an history of (pre)malignancies of the salivary glands. Patients were randomized for parotid-, submandibular- or sham irradiation (2x6 Gy with one week interval). Patients were evaluated at 1, 3, 6 and 12 months after radiation. Primary outcome measure was drooling severity according to the UPDRS. Secondary outcomes measures consisted of stimulated glandular salivary secretion rates and adverse effects.

**Findings:**

Overall 31 parkinsonian patients were included. Initially 11 patients were radiated on the parotid glands, 10 patients on the submandibular glands and 10 patients were sham-radiated. After 6 months, the sham-radiated patients were actively treated after a second randomisation. One patient in the parotid radiation group discontinued his participation after three months due to physical deterioration. Radiation of parotid or submandibular glands significantly improved the existing drooling, as compared to placebo radiation. Parotid- and submandibular radiation was equally effective, but more patients in the submandibular radiated group reported sticky saliva vs. patients treated by parotid radiation (33∙33% vs. 13∙33%).

**Interpretation:**

Major salivary gland radiation significantly improves drooling in parkinsonian patients with few adverse effects. However, parotid gland radiation is accompanied by fewer side effects and therefore is the preferred mode of radiation in this patient population.

## 1 Introduction

Drooling occurs in 32 to 74% of parkinsonian patients, from which the majority is affected by idiopathic Parkinson’s disease. It has a great impact on the quality of life [1], [2], [3], [4]. Patients avoid social activities and interactions, which has a profound impact on the lives of their families too [5], [6], [7]. Drooling is associated with an increased risk of candidiasis, stomatitis and angular cheilitis [8]. Excessive drooling in parkinsonian patients is caused by multiple factors [9]. The most important factor is impaired swallowing of saliva. This causes an overload of saliva, which may result in drooling, eventually worsened by a stooped posture [10], [11], [12]. When saliva is aspirated, it can even result in pneumonia [13]. So, the reduction of drooling in parkinsonian patients is paramount to improve their quality of life and to reduce associated health risks.

Several therapies are available to reduce excessive drooling, but their effectiveness in parkinsonian patients is not well defined [33]. The most widely used therapies are oral anticholinergics, injection of botulin toxin in the major salivary glands, and oral motor training. Seldomly patients get a surgical treatment, removing the salivary glands [14]. In addition, surgical procedures are associated with an increased risk on complications due to the general anesthesia [15]. Also anticholinergics cause problems in parkinsonian patients, because they may worsen cognitive symptoms or induce hallucinations, especially in elderly patients [33], [16], [17], [18]. Botulinum toxin (BTX) injections have some drawbacks as well, f.i. the need for repeated hospital visits, due to the relatively short duration of effect, mostly not exceeding 2–4 months [34], [19], [20], [21]. Oral motor training and/or behavioral therapy are alternative options, which however are impaired frequently by cognitive problems in many PD patients [22].

So, there is a need for an effective and easy to administer therapy for drooling in this patient group. Irradiation of major salivary glands may be helpful, especially if the current therapies are contra-indicated or patients do not respond to these therapies. Earlier studies showed that radiation therapy has the potential to effectively reduce drooling in patients with Parkinson’s disease and amyotrophic lateral sclerosis [23]. These studies reported mild radiation induced side effects, like xerostomia, saliva thickening, taste changes, skin reaction, pain and the very small risk of a radiation induced malignancy [23], [33]. However, no randomized double-blind placebo-controlled studies have been performed so far, which means that radiation therapy is not an established therapy for this indication [23], [24].

Previous studies also did not differentiate between radiation of parotid and submandibular glands [23]. Both glands have shown comparable radiosensitivity, both in animal and men [25], [26]. The submandibular gland saliva is more mucous and is secreted continuously throughout the day, while parotid gland saliva is serous and primarily secreted after stimulation [27]. This fundamental difference could be a key factor in determining the optimal target for radiation.

The aim of this study is to compare the efficacy and safety of submandibular and parotid salivary gland radiation in parkinsonian patients with severe drooling.

### 1.1 Evidence before this study

PubMed was searched for articles published up to December 2020, using the MeSH terms “Parkinson’s disease” and “drooling”, which resulted in 248 articles. Analysis of these articles showed that no studies have been conducted comparing the effect of salivary- and parotid gland radiation, neither any study with sham-radiation.

### 1.2 Added value of this study

This is the first randomised double-blind placebo-controlled clinical trial on irradiation of the major salivary glands to improve drooling in parkinsonian patients. So, this design comparing 3 treatment regimens including placebo, adds value to the existing body of evidence.

### 1.3 Implications of all above evidence

This study shows that radiation of both the parotid- and submandibular glands is effective in reducing drooling in parkinsonian patients. Based on the adverse effects profile, parotid gland radiation is preferable because of the lower incidence of adverse effects, like sticky saliva and dry mouth, compared to radiation of the submandibular glands.

## 2 Methods

### 2.1 Study design

This placebo-controlled, double-blind, prospective, randomized clinical trial was performed at the University Medical Center of Groningen (UMCG) in the Netherlands. The study was approved by the ethical review board of the UMCG. The study was supported by a grant of the Beatrix fund in the Netherlands

### 2.2 Patients

Patients with a clinical diagnosis of parkinsonism and a history of severe drooling were assessed at the neurology department of the UMCG. The Unified Parkinson’s Disease Rating Scale (UPDRS), especially item 6, assessing severity of drooling, was used as primary endpoint [28]. Patient’s with a score of ≥ 3 on this item (marked excess of saliva with some drooling) could be included in the study. The Hoehn and Yahr scale was used to rate the baseline severity of parkinsonism [29]. Patients were excluded if they participated at the same time in another investigational study, used anticholinergic drugs, had previously surgical procedures in the oral or nasal cavity that might affect salivary secretion, or had an history of (pre)malignancies in the radiation area (Table 1).

**Table 1:** Baseline demographics and clinical characteristics (means with SD or ranges).

	Placebo (n = 10)	Parotid (n = 11)	Submandibular (n = 10)

Sex			
Male	10	8	7
Female	0	3	3
Age (years)	68∙5 (7∙37) SD	69∙2 (7∙03) SD	67∙4 (10∙11) SD
Score on item 6 of the ADL-section of the UPDRS			
3	4	6	6
4	6	5	4
Duration of disease (years)	11∙5 (4–22) min/max	13∙5 (4–29) min/max	9∙4 (4–24) min/max
Hoehn and Yahr score			
1		1	
2	1	1	1
3	6	5	6
4	3	4	2
5			1
Previous treatment			
Anticholinergic drug	5	3	3
Botulinum toxin			1
Radiation therapy	3		
Quality of life assessment			
Choking on saliva	8	6	4
Use of handkerchief	10	11	10
Feeling of limitation in social activities due to drooling	5	7	6

### 2.3 Randomisation and masking

The included patients were randomized across 3 arms: sham-radiation, radiotherapy (RT) of the parotid glands or RT of the submandibular glands. After six months all patients from the sham-group were also radiated, randomly assigned to parotid- or submandibular gland RT.

### 2.4 Procedures

All patients received an individual thermoplastic five points head, neck and shoulder immobilisation mask to fixate the head during acquisition of the planning CT-scan and the radiation. The planning CT-was acquired, extending from the top of the skull to the clavicle, for all patients. This planning CT-scan was used to define the exact location of the parotid- and submandibular glands. The prescribed radiation dose was 12 Gy in two fractions of 6 Gy, with an interval of one week. Radiotherapy to both the parotid glands comprised electrons, (14 MeV, the dose prescribed at Dmax) which were administered by a linear accelerator. Sham-radiated patients followed the same procedure, except from the actual radiation. Radiotherapy to both submandibular glands for most patients also compromised electrons (Range 10–14 MeV, prescribed at D-max, depending on the located depth of the submandibular glands). In 2 patients radiotherapy with electrons was not possible at the submandibular glands. The radiation tube used for electrons could not be placed appropriately in these patients, who received 6 MV photons thereafter. Both radiation schedules resulted in a comparable radiation volume and a cumulative radiation dose to the target salivary gland tissue.

### 2.5 Follow-up and evaluation

All groups had assessments at baseline, 1, 3, and 6 months by a blinded rater. Only patients in the initial parotid-RT or submandibular-RT groups were assessed during 12 months . Patients in the placebo-RT group received actual radiation of either their parotid (n = 5) or submandibular glands (n = 5) after 6 months.

The primary endpoint consisted of the score on item 6 of the UPDRS. Stimulated salivary flow, (both submandibular- and parotid glands) served as secondary outcome measures, as well as the assessment of adverse events using a standardised structured adverse event questionnaire.

The score on item 6 of the UPDRS was rated by the patients. This score consists of five degrees of drooling severity, ranging from 0 to 4, whereas 4 represents severe drooling with the need for tissues.

The secondary outcome was the stimulated salivary flow of the parotid and submandibular/sublingual glands. Saliva of the parotid glands was collected by pre-weighted gauzes placed in the mouth at the orifices of the parotid duct. The submandibular/sublingual saliva was collected every 30 s using a monoject®, which was placed at the floor of mouth and stored in a closable pre-weighed tube. Saliva was collected during 10 min, stimulated by citric acid, which was dripped on the tongue every minute. After 10 min the weight of gauze sponges and monojects was measured again [35].

At baseline, sex and age were registered, as well as data on disease duration and Hoehn and Yahr scores. Previous treatments for drooling were recorded as well. Quality of life was assessed by questions on choking on saliva, the use of handkerchief’s and the feeling of being limited in social activities’ due to drooling.

Adverse events (AE) were measured by asking the patients whether they experienced any adverse effects during the course of treatment and follow-up. If AE were reported, the severity was rated by the patients, using a 3- point scale (mild, moderate, severe). Adverse events were rated at every follow-up visit.

### 2.6 Statistical analysis

The power-analysis was based on previously RT data in patients with drooling [23]. To reach a power of 95% with an alpha of 0∙05 it was calculated that 30 patients should be included in this study. All the analyses were performed with the use of SPSS 22.0. Analyses were performed separately for each, follow-up moment, up to 12 months for the patients initially randomized for RT. Differences between groups in perceived drooling and quantitative salivary flow were analysed using ANCOVA with baseline score as a covariate. Wilcoxon signed rank tests were used to compare the differences on item 6 of the ADL-section of the UPDRS between all groups. Adverse events were actively collected and rated at all pre-defined follow-up moments.

### 2.7 Role of the funding source

Funding for this study was provided by the Dutch Beatrix Fund. The funding institute had no role in study design, data collection, data analysis, data interpretation, or writing of the report.

## 3 Results

**Fig. 1:**
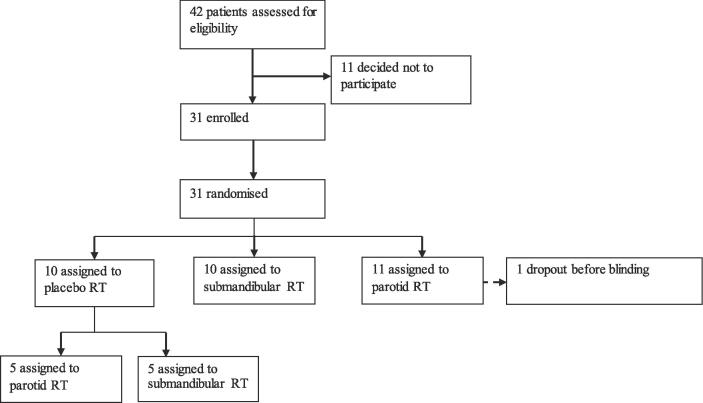
Study design.

Overall 42 patients were selected. Thirty-one patients signed the informed consent and were included in the study. Eleven patients were not included because they refused radiation (n = 7) or withdrew because of travel distance (n = 4) (Fig. 1)

Baseline characteristics are shown in table 1. All patients were diagnosed with Parkinson’s disease (PD), based on the clinical examination of the UMCG neurologists, except one, who was diagnosed with Lewy Body Dementia, which is a variant of PD (placebo-RT group). None of the patients had severe dysphagia needing a gastric tube. One subject from the parotid-RT group terminated the study prematurely due to physical deterioration, not related to RT. This patient has been replaced by an extra patient in the parotid-RT group. One subject died before the 12 months follow up, also not related to RT.

### 3.1 Rating of the salivary flow burden by patients (UPDRS-6)

**Fig. 2:**
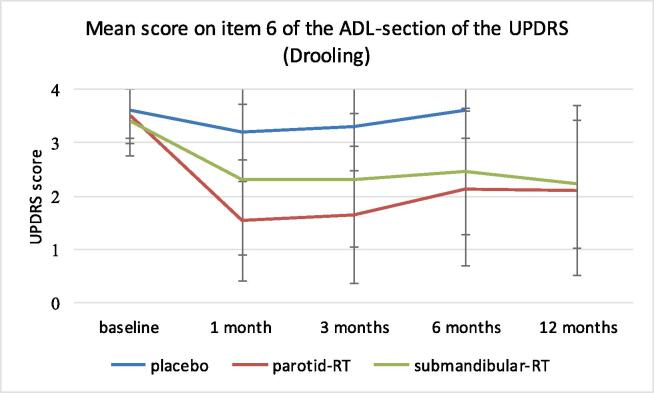
Mean scores with standard deviation on item 6 of the ADL-section of the UPDRS over time.

Fig. 2 shows the scores on item 6 of the ADL-section of the UPDRS (UPDRS-6) for all groups over time. The parotid-RT group showed a significant reduction of the UPDRS-6 score vs. placebo after 1 month (p < 0∙000), which was not the case in the submandibular-RT group (p = 0∙183). This significant difference vs. placebo was maintained at 3 months in the parotid-RT group (p = 0∙001) with a trend for significant improvement in the submandibular-RT group (p = 0∙084). At six months the UPDRS- 6 score vs. placebo was significantly reduced in both the parotid-RT- (p < 0∙000) as well as the submandibular-RT group (p = 0∙011). Both treatment groups did not differ significantly at 6 months (p = 0∙250) and at 12 months (p = 0∙689).

### 3.2 Stimulated salivary flow

**Fig. 3:**
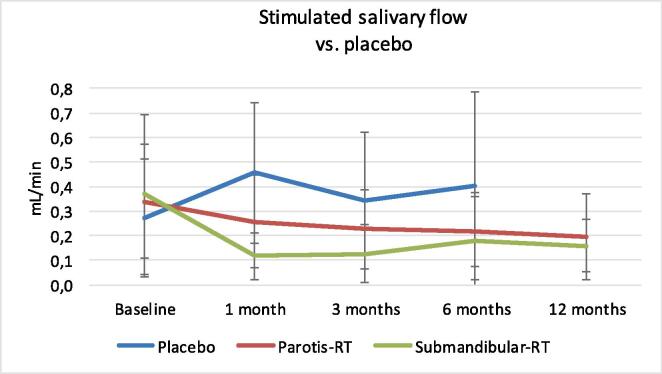
Effect of radiation therapy and placebo-radiation on the stimulated salivary flow as a function of time in ml/min (mean scores with standard deviation).

At one month the submandibular-RT group (p = 0·001) and the parotid-RT group (p = 0·016) both showed a significant reduction of the stimulated salivary flow compared to placebo (Fig. 3). At three months only the submandibular-RT group still had a significant reduction in salivary flow (p = 0·007). At six months both the submandibular- and parotid salivary flow were significantly reduced vs. placebo (submandibular-RT group (p = 0·004), parotid-RT group (p = 0·009). No significant difference was found between the submandibular-RT and parotid-RT groups at 6 months (p = 0·510) and 12 months (p = 0·833).

### 3.3 Adverse events

**Fig. 4:**
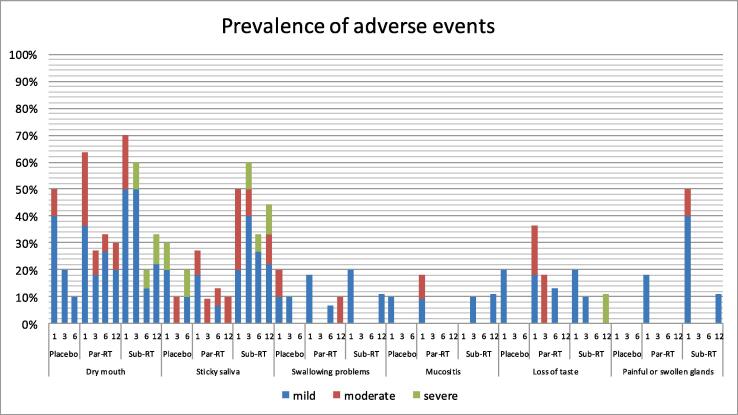
Prevalence of adverse events (%). Adverse events were recorded at 1, 3, 6 and 12 months after start of radiotherapy.

Fig. 4 shows the relative number of adverse events in all groups. All adverse events were rated according a 3-point scale of severity (mild-moderate-severe). Dry mouth, sticky saliva, swallowing problems, mucositis, loss of taste and painful or swollen glands were reported most frequently. However, after three months most adverse events had improved. Sticky saliva and dry mouth were seen more frequently in the submandibular-RT group compared to the parotid-RT group.

## 4 Discussion

Our study shows that radiotherapy on either the parotid or submandibular glands with 2 fractions of 6 Gy with one week interval, significantly improves drooling in patients with parkinsonism, both subjectively and objectively. The effect lasted for at least 12 months in our population. Radiation of the parotid gland was as effective as radiation of the submandibular glands, but parotid-RT showed an earlier effect onset and had less adverse effects. Therefore, the parotid glands should be the primary target for radiotherapy in case of sialorhoea in patients with parkinsonism.

This subjective improvement, as scored by the UPDRS-item 6 self-assessment, was supported by an objective reduction of the parotid- and submandibular stimulated salivary flow, which was still present 12 months post-radiation.

Most of the reported adverse effects were transient. However, sticky saliva and dry mouth were reported most frequently and lasted for several weeks. Sticky saliva was reported more frequently in the submandibular group, which could be due to the constantly secreted mucous saliva, produced by the submandibular gland [27].

Reliable saliva collection is difficult in patients suffering from parkinsonism [30]. The nature of the underlying disease makes it impossible to obtain sheer unstimulated saliva, due to hyperkinetic head-movements, whereas also the gauze sponges placed in the mouth may stimulate salivary flow. [31]

At the final follow-up at 12 months, 5 patients were not satisfied about the final effect of radiotherapy on their sialorhoea (3 after submandibular-RT and 2 after parotid-RT). They received an additional RT dose of 12 Gy (2 times 6 Gy) on their salivary glands, which resulted in a satisfying reduction of their salivary flow in all cases. So, the radiation dose can be increased on an individual basis above 12 Gy if the effect is insufficient, as long as the radiation safety threshold of 30 Gy is not bypassed [23].

This study is the first randomized placebo-controlled trial of radiotherapy for parkinsonian patients with excessive drooling, with an adequate follow-up. Earlier studies had limited study designs and did not involve parotid and submandibular gland radiation [23], [24].

For the primary endpoint we used the UPDRS-item 6 self-assessment, which was common when the study was designed. In other studies investigating other current therapies the Teacher drooling scale is used [33], [34]. For potential future studies we will also incorporate the Teacher drooling scale.

For this study, we used the radiation schedule of 2 times 6 Gy, which is based on a previous study [36]. Several other studies concerning various neurological conditions used different radiation schedules. [33] In a study, concerning only patients with amyotrophic lateral sclerosis (ALS), 20 Gy given in 4 fracties was also safe and effective [37]. The most optimal radiation schedule and dosage should investigated in future studies to balance between effectiveness and side effects.

For this study, patients had to lay down on the radiation table with an immobilisation mask. This was needed to precisely define the different salivary glands on CT and radiate them. Fortunately, all patient did tolerate this procedure. For some patients, laying down might be very cumbersome. For these patients orthovolt radiation given in sitting position might be an alternative.

Radiation of the salivary glands might have benefits as compared to the current other therapies. Anticholinergic medication is able to reduce sialorhoea [33]. Especially, glycopyrrolate has been proven to be effective several studies, which can be easily administered and has less central nervous side effects compared to other anticholinergic medications. Still, due to the numerous side effects and contraindications anticholinergics are often not suitable for patients with advanced parkinsonism [4], [32], [33]. Speech therapy has only a limited effect, especially during the early stages of parkinsonism, and has to be continued, which is a burden for the patients [20]. Botulinum toxin (BTX) is an effective and save therapy [34]. Most BTX related side effects, like xerostomia, are mild to moderate in severity and usual self-limiting [34]. Unfortunately, the effect of BTX only lasts for approximal 3 months, which makes repeated injections necessary, also providing a burden for the patients. [17]

In 2016, Weikamp, et al [38] performed a prospective randomize controlled pilot study in which 10 patients with ALS received RT and 10 patients with received BTX. The RT was given on both parotid glands and the posterior part of the submandibular gland using a single dose of 7 Gy. The BTX (botulinum neurotoxin serotype A) was injected only in the parotid gland with low dose of 50 MU. The study did not find significant differences between the two treatment arms concerning the burden of drooling. However, at twelve weeks after treatment more saliva reduction was achieved with RT compared to BTX. Possibly confirming the more long-lasting effect of RT as seen in our study.

In conclusion, major salivary gland radiation significantly improves drooling in parkinsonian patients with few adverse effects. Parotid gland radiation is accompanied by fewer side effects and therefore is the preferred mode of radiation in this patient population. Future studies should focus on RT comparing with other different treatment options (especially BTX) to evaluate which treatment is most effective, less burdensome and gives the best quality of life.

## CRediT authorship contribution statement

**R.J.H.M. Steenbakkers:** Methodology, Investigation, Writing – review & editing, Supervision, Project administration. **S.P. van Doornik:** Formal analysis, Investigation, Writing – original draft, Project administration. **A. Vissink:** Methodology, Writing – review & editing. **W. Kerdijk:** Formal analysis, Writing – review & editing. **T. van Laar:** Conceptualization, Methodology, Writing – review & editing, Supervision.

## Declaration of Competing Interest

The authors declare that they have no known competing financial interests or personal relationships that could have appeared to influence the work reported in this paper.

## References

[1] Kalf J.G., Swart B.J.M., Borm G.F., Bloem B.R., Munneke M. (2009). Prevalence and definition of drooling in Parkinson's disease: a systematic review. J. Neurol..

[2] Kalf J.G., Smit A.M., Bloem B.R., Zwarts M.J., Munneke M. (2007). Impact of drooling in Parkinson's disease. J. Neurol..

[3] Leibner J., Ramjit A., Sedig L., Dai Y., Wu S.S., Jacobson C., Okun M.S., Rodriguez R.L., Malaty I.A., Fernandez H.H. (2010). The impact of and the factors associated with drooling in Parkinson's disease. Parkinsonism Relat. Disord..

[4] Molloy L. (2007). Treatment of sialorrhoea in patients with Parkinson's disease: best current evidence. Curr. Opin. Neurol..

[5] Srivanitchapoom P., Pandey S., Hallett M. (2014). Drooling in Parkinson's disease: a review. Parkinsonism Relat. Disord..

[6] Ozdilek B., Gunal D.I. (2012). Motor and non-motor symptoms in Turkish patients with Parkinson's disease affecting family caregiver burden and quality of life. J. Neuropsychiatry Clin. Neurosci..

[7] Squires N., Wills A., Rowson J. (2012). The management of drooling in adults with neurological conditions. Curr. Opin. Otolaryngol. Head Neck Surg..

[8] Meningaud J.-P., Pitak-Arnnop P., Chikhani L., Bertrand J.-C. (2006). Drooling of saliva: a review of the etiology and management options. Oral Surg. Oral Med. Oral Pathol. Oral Radiol. Endod..

[9] Chou K.L., Evatt M., Hinson V., Kompoliti K. (2007). Sialorrhea in Parkinson's disease: a review. Mov. Disord..

[10] Kalf J.G., Munneke M., van den Engel-Hoek L., de Swart B.J., Borm G.F., Bloem B.R., Zwarts M.J. (2011). Pathophysiology of diurnal drooling in Parkinson's disease. Mov. Disord..

[11] Nicaretta D.H., Rosso A.L., Mattos J.P.d., Maliska C., Costa M.M.B. (2013). Dysphagia and sialorrhea: the relationship to Parkinson's disease. Arq. Gastroenterol..

[12] Bakke M., Larsen S.L., Lautrup C., Karlsborg M. (2011). Orofacial function and oral health in patients with Parkinson’s disease. Eur. J. Oral Sci..

[13] Nobrega A.C., Rodrigues B., Melo A. (2008). Is silent aspiration a risk factor for respiratory infection in Parkinson’s disease patients?. Parkinsonism Relat. Disord..

[14] Reed J., Mans C.K., Brietzke S.E. (2009). Surgical management of drooling: a meta-analysis. Arch. Otolaryngol. Head Neck Surg..

[15] Nicholson G., Pereira A.C., Hall G.M. (2002). Parkinson's disease and anaesthesia. Br. J. Anaesth..

[16] Duvoisin R.C. (1967). Cholinergic-anticholinergic antagonism in parkinsonism. Arch. Neurol..

[17] Meco G., Casacchia M., Lazzari R. (1984). Mental impairment in Parkinson's disease. The role of anticholinergic drugs. Acta Psychiatr. Belg..

[18] Koller W.C. (1984). Disturbance of recent memory function in parkinsonian patients on anticholinergic therapy. Cortex.

[19] Truong D.D., Jost W.H. (2006). Botulinum toxin: clinical use. Parkinsonism Relat. Disord..

[20] Narayanaswami P., Geisbush T., Tarulli A., Raynor E., Gautam S., Tarsy D., Gronseth G. (2016). Drooling in Parkinson's disease: a randomized controlled trial of incobotulinum toxin A and meta-analysis of Botulinum toxins. Parkinsonism Relat. Disord..

[21] Tan E.K., Lo Y.L., Seah A., Auchus A.P. (2001). Recurrent jaw dislocation after botulinum toxin treatment for sialorrhoea in amyotrophic lateral sclerosis. J. Neurol. Sci..

[22] Marks L., Turner K., O'sullivan J., Deighton B., Lees A. (2001). Drooling in Parkinson's disease: a novel speech and language therapy intervention. Int. J. Lang. Commun. Disord..

[23] Hawkey N.M., Zaorsky N.G., Galloway T.J. (2016). The role of radiation therapy in the management of Sialorrhea: a systematic review. Laryngoscope.

[24] Potulska A., Friedman A. (2005). Controlling sialorrhoea: a review of available treatment options. Expert Opin. Pharmacother..

[25] Coppes R.P., Vissink A., Konings A.W.T. (2002). Comparison of radiosensitivity of rat parotid and submandibular glands after different radiation schedules. Radiother. Oncol..

[26] Konings A.W.T., Coppes R.P., Vissink A. (2005). On the mechanism of salivary gland radiosensitivity. Int. J. Radiat. Oncol. Biol. Phys..

[27] Humphrey S.P., Williamson R.T. (2001). A review of saliva: normal composition, flow, and function. J. Prosthet. Dent..

[28] Goetz C.G., Fahn S., Martinez-Martin P., Poewe W., Sampaio C., Stebbins G.T., Stern M.B., Tilley B.C., Dodel R., Dubois B., Holloway R., Jankovic J., Kulisevsky J., Lang A.E., Lees A., Leurgans S., LeWitt P.A., Nyenhuis D., Olanow C.W., Rascol O., Schrag A., Teresi J.A., Van Hilten J.J., LaPelle N. (2007). Movement Disorder Society-sponsored revision of the Unified Parkinson's Disease Rating Scale (MDS-UPDRS): process, format, and clinimetric testing plan. Mov. Disord..

[29] Hoehn M.M., Yahr M.D. (1967). Parkinsonism: onset, progression and mortality. Neurology.

[30] Mulligan R., Navazesh M., Wood G.J. (1995). A pilot study comparing three salivary collection methods in an adult population with salivary gland hypofunction. Spec. Care Dentist..

[31] Navazesh M. (1993). Methods for collecting saliva. Ann. N. Y. Acad. Sci..

[32] Mier R.J., Bachrach S.J., Lakin R.C., Barker T., Childs J., Moran M. (2000). Treatment of sialorrhea with glycopyrrolate: a double-blind, dose-ranging study. Arch. Pediatr. Adolesc. Med..

[33] Steffen A., Jost W., Bäumer T., Beutner D., Degenkolb-Weyers S., Groß M., Grosheva M., Hakim S., Kahl K.G., Laskawi R., Lencer R., Löhler J., Meyners T., Rohrbach-Volland S., Schönweiler R., Schröder S.-C., Schröder S., Schröter-Morasch H., Schuster M., Steinlechner S., Urban R., Guntinas-Lichius O. (2019). Hypersalivation: update of the German S2k guideline (AWMF) in short form. J. Neural Transm..

[34] Yu Y.-C., Chung C.-C., Tu Y.-K., Hong C.-T., Chen K.-H., Tam K.-W., Kuan Y.-C. (2022). Efficacy and safety of botulinum toxin for treating sialorrhea: A systematic review and meta‐analysis. J. Neurol..

[35] Vissink A., Wolff A., Veerman E.C.I., Wong D.T. (2008). Saliva diagnostics.

[36] Postma A.-G., Heesters M., van Laar T. (2007). van Laar T (2007) Radiotherapy to the salivary glands as treatment of sialorrhea in patients with parkinsonism. Mov. Disord..

[37] Assouline A., Levy A., Abdelnour-Mallet M., Gonzalez-Bermejo J., Lenglet T., Le Forestier N., Salachas F., Bruneteau G., Meininger V., Delanian S., Pradat P.-F. (2014). Pradat PF (2014) Radiation therapy for hypersalivation: a prospective study in 50 amyotrophic lateral sclerosis patients. Int. J. Radiat. Oncol. Biol. Phys..

[38] Weikamp J.G., Schinagl D.A.X., Verstappen C.C.P., Schelhaas H.J., de Swart B.J.M., Kalf J.G. (2016). Botulinum toxin-A injections vs radiotherapy for drooling in ALS. Acta Neurol. Scand..

